# Effect of lower body negative pressure on cardiac and cerebral function in postural orthostatic tachycardia syndrome: A pilot MRI assessment

**DOI:** 10.14814/phy2.15979

**Published:** 2024-03-15

**Authors:** Rachel J. Skow, Stephen J. Foulkes, Peter Seres, Meghan A. Freer, Eric D. Mathieu, Satish R. Raj, Richard B. Thompson, Mark H. Haykowsky, Lawrence Richer

**Affiliations:** ^1^ Integrated Cardiovascular Exercise Physiology and Rehabilitation (iCARE) Laboratory, College of Health Sciences University of Alberta Edmonton Alberta Canada; ^2^ Department of Radiology and Diagnostic Imaging University of Alberta Edmonton Alberta Canada; ^3^ Women and Children's Health Research Institute University of Alberta Edmonton Alberta Canada; ^4^ Department of Cardiac Sciences, Libin Cardiovascular Institute, Cumming School of Medicine University of Calgary Calgary Alberta Canada; ^5^ Department of Biomedical Engineering University of Alberta Edmonton Alberta Canada; ^6^ Department of Pediatrics University of Alberta Edmonton Alberta Canada

**Keywords:** arterial spin labelling, cardiac function, cerebral blood flow, magnetic resonance imaging, orthostatic intolerance

## Abstract

Postural orthostatic tachycardia syndrome (POTS) is characterized by an excessive heart rate (HR) response upon standing and symptoms indicative of inadequate cerebral perfusion. We tested the hypothesis that during lower body negative pressure (LBNP), individuals with POTS would have larger decreases in cardiac and cerebrovascular function measured using magnetic resonance (MR) imaging. Eleven patients with POTS and 10 healthy controls were studied at rest and during 20 min of −25 mmHg LBNP. Biventricular volumes, stroke volume (SV), cardiac output (Qc), and HR were determined by cardiac MR. Cerebral oxygen uptake (VO_2_) in the superior sagittal sinus was calculated from cerebral blood flow (CBF; MR phase contrast), venous O_2_ saturation (SvO_2_; susceptometry‐based oximetry), and arterial O_2_ saturation (pulse oximeter). Regional cerebral perfusion was determined using arterial spin labelling. HR increased in response to LBNP (*p* < 0.001) with no group differences (HC: +9 ± 8 bpm; POTS: +13 ± 11 bpm; *p* = 0.35). Biventricular volumes, SV, and Qc decreased during LBNP (*p* < 0.001). CBF and SvO_2_ decreased with LBNP (*p* = 0.01 and 0.03, respectively) but not cerebral VO_2_ (effect of LBNP: *p* = 0.28; HC: −0.2 ± 3.7 mL/min; POTS: +1.1 ± 2.0 mL/min; *p* = 0.33 between groups). Regional cerebral perfusion decreased during LBNP (*p* < 0.001) but was not different between groups. These data suggest patients with POTS have preserved cardiac and cerebrovascular function.

## 1 INTRODUCTION

Postural orthostatic tachycardia syndrome (POTS) is a syndrome that is characterized not only by the excessive postural mediated tachycardia but also by prominent and long‐standing symptoms associated with orthostatic intolerance (e.g., light‐headedness, headache, nausea, and cognitive deficits) while upright that are relieved by recumbence (Stewart, 2013). The estimated prevalence overall of POTS is up to 1.0% of the population with a peak onset in mid to late adolescence and 75% of cases occurring in young females (Johnson et al., 2010). This syndrome can be very debilitating with measures of quality of life, sleep, and activities of daily living often greatly impacted (Benrud‐Larson et al., 2002). The underlying causes of, and relationships between, the defining symptoms of POTS have been attributed to decreased cardiac output (Qc) and/or cerebral blood flow (CBF) (Duschek et al., 2009; Ogoh, 2017; Ogoh & Tarumi, 2019); however, this has not been definitively established.

Previously, cardiac factors linked to POTS include lower stroke volume (SV) and Qc associated with a smaller heart (Fu et al., 2010). Patients with POTS not only increase their heart rate (HR) to a greater extent when compared to healthy individuals (as defined by their diagnosis), but also experience decreases in SV that appears to be related to the magnitude of increase in HR (Masuki et al., 2010). In response to standing, they also exhibit altered cardiovascular responses including a greater incidence of orthostatic hypotension (Stewart et al., 1985). However, not all studies have reported a differential reflexive decrease in Qc upon standing between patients with POTS and healthy controls (Fu et al., 2010). Therefore, it is not evident whether the differences in cardiac function at rest result in altered cardiac responsivity to orthostatic challenge.

It is also hypothesized that during orthostatic challenge, differences in the cerebrovascular response may emerge between patients with POTS and healthy controls. Indeed, cerebral tissue saturation measured by oximetry was previously reported to be lower in patients with POTS upon standing (Kharraziha et al., 2019). However, studies using transcranial Doppler ultrasound to measure cerebral blood velocity have yet to demonstrate differences at rest or in response to orthostatic challenge between the two groups (Gelpi et al., 2023; Lin et al., 2020; Stewart et al., 2015; Stewart & Medow, 1985; Wells et al., 2020). To date, measures of cerebrovascular function using magnetic resonance imaging (MRI) have not been performed in this population and may provide additional mechanistic insight due to the ability to measure CBF, regional perfusion, and oxygen uptake (e.g., cerebral VO_2_).

Lastly, we do know that upright posture is associated with decreased cognitive function in patients with POTS (Ocon et al., 2012; Stewart et al., 2012). During a prolonged cognitive stress test, patients with POTS have a greater reduction in cerebral blood velocity that corresponds with a greater symptom score (Wells et al., 2020). Taken together, these data suggest that altered cerebrovascular responsiveness may play a role in symptomology in patients with POTS. Therefore, the aim of this study was to use MRI to test the hypothesis that there would be a greater reduction in Qc (and its determinants), CBF and perfusion, and cognitive function during lower body negative pressure (LBNP) in patients with POTS compared to healthy age‐ and sex‐matched controls.

## 2 METHODS

Eleven patients with POTS (age: 17 ± 1 years; 1 male) and 10 healthy controls (18 ± 2 years; 1 male) were enrolled. Participants with POTS were recruited through an autonomic function clinic at the Stollery Children's Hospital run by the study investigator (LR) and met the diagnostic criteria for POTS based on standardized tilt table test or active stand test performed in the clinic (Raj et al., 2020). They also completed an autonomic symptom assessment and reported at least two or more symptoms commonly associated with POTS on most days, for more than 3 months. Healthy control participants were screened using an active standing test and were excluded if their HR increased >30 bpm. All participants provided written consent or assent (if <18 years) prior to participating.

All participants were asked to refrain from taking their regular medications for five half‐lives (3–4 days) except for contraceptives and any other stable medications deemed medically necessary that do not influence the autonomic nervous system or cardiac autonomic reflexes. Additionally, all participants were asked to refrain from all intake of xanthine‐, caffeine‐, or alcohol‐containing substances for 72 h prior to the assessment.

Participants were sealed into a custom built, MR‐compatible LBNP chamber (Esch et al., 2010) from the iliac crest down and placed headfirst into a 3T Siemens PRISMA MRI scanner (Erlangen, Germany). HR and arterial oxygen saturation (SpO_2_; %) were monitored continuously throughout (pulse oximeter) and blood pressure (BP; automated brachial cuff) was taken every 5 min during the protocol. Reported values for resting HR, BP, and SpO_2_ were determined as the average of three values. Mean arterial BP was calculated as 1/3 systolic BP + 2/3 diastolic BP. Cardiac and cerebral outcomes were measured at rest and after at least 10 min of continual LBNP at −25 mmHg and measure concomitant changes in cardiac function and cerebral oxygenation and perfusion.

**FIGURE 1:**
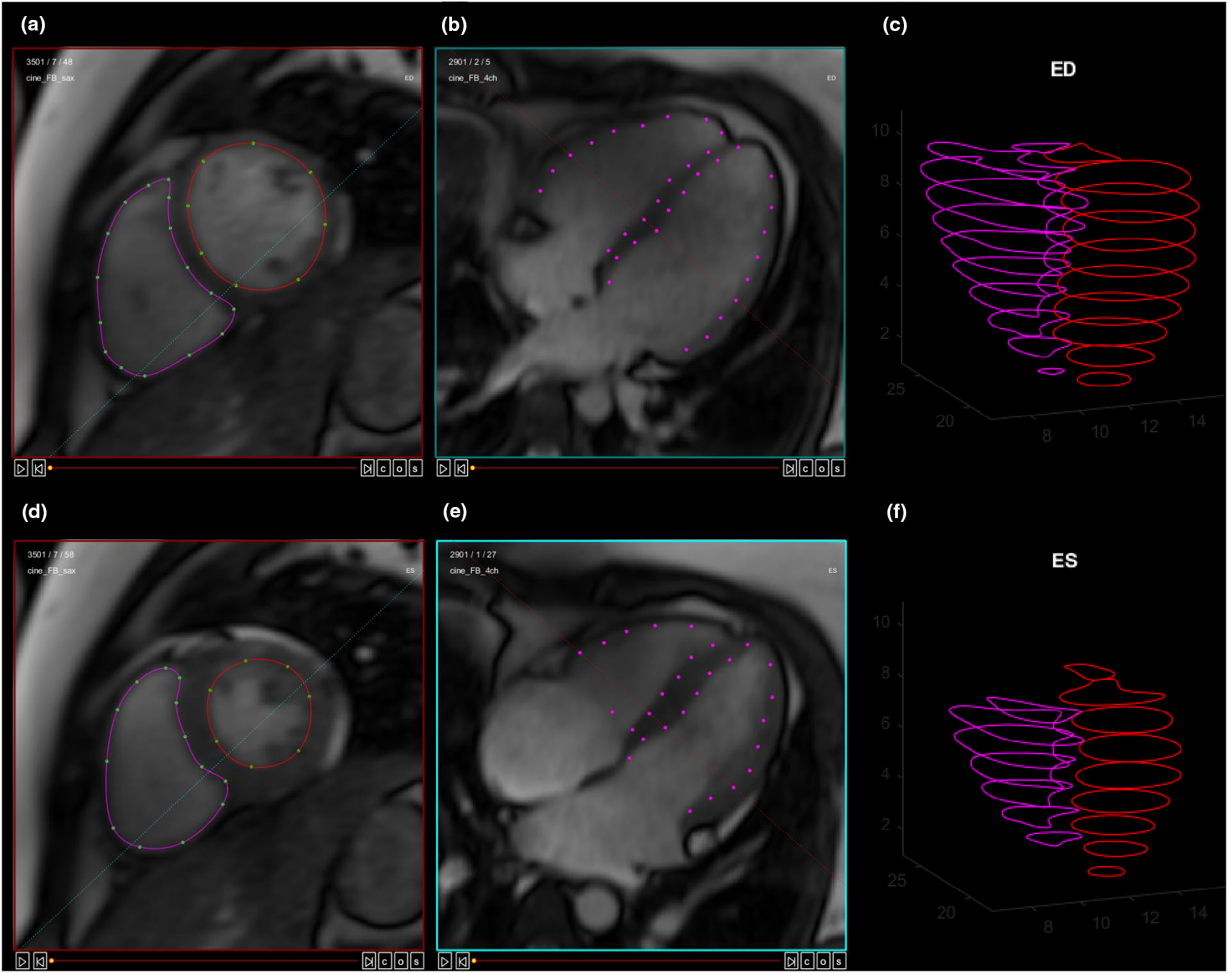
Example of real‐time ungated free‐breathing cardiac MRI imaging. Endocardial borders of the left (red contours) and right ventricle (pink contours) were traced on short‐axis images at (a) end‐diastole (ED) and (d) end‐systole (ES). Transection of the short‐axis contours with the horizontal long‐axis plane for the corresponding phase of the cardiac cycle (b, e) is shown by the pink dots. Areas for each short‐axis slice at ED (c) ED and (f) ES are summed using the summation of discs method to determine ED and ES ventricular volumes.

**FIGURE 2:**
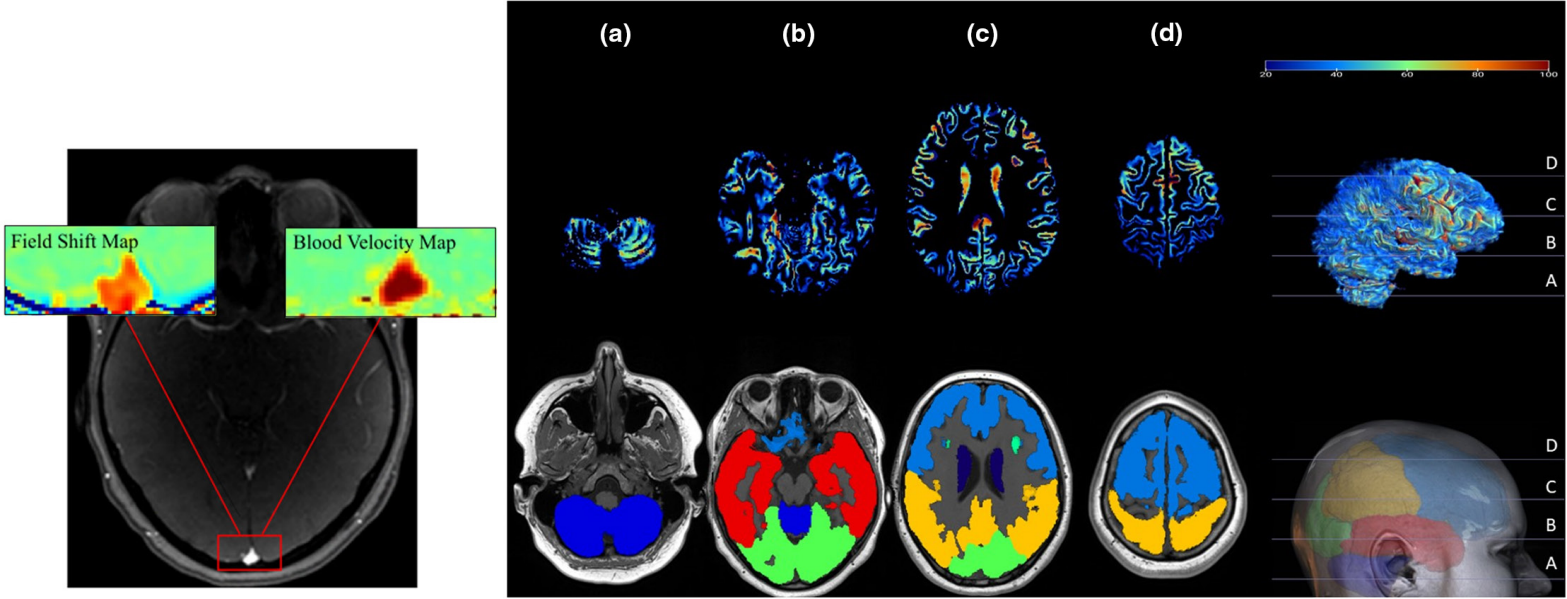
(Left) Representation of simultaneous measurement of venous oxygen saturation (left inset) and cerebral blood flow (right inset) in the superior sagittal sinus (red outline at bottom of image). (Right) Arterial spin labelling determination for estimation of regional cerebral perfusion in four representative imaging slices (top, a–d) and color maps (bottom) representing distinct brain regions (light blue, frontal lobe; green, occipital lobe; yellow, parietal lobe; red, temporal lobe; and dark blue, cerebellum).

Cardiac function was determined as left and right ventricular end‐diastolic and end‐systolic volume (EDV and ESV, respectively; Figure 1), SV, and Qc (SV × HR). Cardiac volumes were measured during real‐time free‐breathing image acquisition as previously described (Beaudry et al., 2018; Esch et al., 2010; Thompson et al., 2016). Cerebral venous oxygen saturation (SvO_2_) and CBF were measured in the superior sagittal sinus **(**Figure 2) and used to calculate cerebral oxygen utilization (VO_2_) as CBF × [(SpO_2_ × Hgb × 1.34) – (SvO_2_ × Hgb × 1.34)], where Hgb is hemoglobin. Hgb and hematocrit were assumed to be 13.5 g/dL and 42% or 15.0 g/dL and 45%, for females and males, respectively, based on normative values for adults (Fulgoni 3rd et al., 2019; Mahlknecht & Kaiser, 2010). Regional cerebral perfusion in all gray matter, the cerebellum, and separately for the frontal, occipital, parietal, and temporal lobes was determined using arterial spin labelling (Figure 2). Detailed MRI methods can be found in the *Online Supplement*.

Cognitive function was assessed by a visual two‐back test, which consisted of a series of 20–22 targets given over a 1‐minute period performed during rest and LBNP. Mean response time and the percentage of correct answers were used as performance metrics. The Vanderbilt Orthostatic Symptom Score (VOSS) was also obtained from all participants at rest and during LBNP.

All data are presented as mean ± SD, unless otherwise specified. Group characteristics and resting data were compared using an unpaired, two‐tailed *t*‐test and data were tested for normality using the Shapiro–Wilk's test. If data were not normally distributed, they are presented as median (95% confidence interval) and the groups were compared using a nonparametric Mann–Whitney *U*‐test. Two‐factor repeated measures analysis of variance was used to determine the effect of LBNP and assess whether the responses to LBNP were different between groups (i.e., interaction effect). The sample size was determined based on a difference between means in cerebral perfusion of 10 mL/100grams during LBNP (calculated from pilot data collected by our research group) and based on previous work by our group showing differential cardiac responses to LBNP in endurance athletes compared to sedentary controls where we had 8 patients per group (Esch et al., 2010). Pearson product moment correlation coefficients were determined to measure the relationships between VOSS during LBNP and outcome measures in the POTS group. All statistics were performed using GraphPad Prism (v10.0.2). Significance was set a‐priori at *p* < 0.05.

## 3 RESULTS

Patients with POTS were similar in age, height, weight, and body mass compared to healthy controls (Table 1). Patients were diagnosed 3 ± 2 years prior to the study assessment and had been experiencing symptoms for 6 ± 4 years. All patients experienced symptoms of orthostatic intolerance ranging from mild to severe. The most common symptoms were dizziness or light‐headedness (with or without a loss of consciousness; *n* = 8), fatigue or tiredness (*n* = 5), and headaches or migraines (*n* = 5). Seven of the participants with POTS (64%) also had family members with a history of symptoms of autonomic dysfunction. Patients with POTS were taking medications for their low BP (*n* = 7), allergies (*n* = 2), headaches (*n* = 2), gastrointestinal issues (*n* = 3), asthma (*n* = 2), thyroid (*n* = 1), Type 1 diabetes (*n* = 1), and mental health (attention deficit disorder, depression, or generalized anxiety disorder; *n* = 5). The participants in the control group were healthy, did not experience tachycardia upon standing, and none reported any medication use.

**TABLE 1:** Participant characteristics, cardiovascular measures, and MRI‐derived measures for cardiac and cerebral function.

	Control (*n* = 10)	POTS (*n* = 11)	*p*‐value
Characteristics			
Age (years)	18 ± 2	18 ± 1	0.39
Height (cm)	168.6 ± 7.3	167.4 ± 8.5	0.74
Weight (kg)	69 (58–81)	57 (53–76)	0.38
BMI (kg/m^2^)	24.4 (20.1–28.9)	19.9 (19.2–27.0)	0.38
BSA (m^2^)	1.8 ± 0.2	1.8 ± 0.2	0.51
Resting cardiovascular measures			
HR (bpm)	74 ± 10	79 ± 13	0.31
Systolic BP (mmHg)	106 ± 8	102 ± 7	0.22
Diastolic BP (mmHg)	64 ± 8	63 ± 5	0.66
Mean arterial BP (mmHg)	78 ± 7	76 ± 5	0.38
Oxygen saturation (SpO_2_; %)	97 ± 2	98 ± 1	0.36
Resting cardiac measures^a^			
Left ventricular EDV (mL)	148 ± 35	129 ± 28	0.19
Left ventricular ESV (mL)	59 (54–78)	54 (48–68)	0.18
Left ventricular EF (%)	55 ± 4	55 ± 4	0.85
Right ventricular EDV (mL)^b^	147 ± 38	118 ± 28	0.09
Right ventricular ESV (mL)^b^	69 ± 20	56 ± 15	0.13
Right ventricular EF (%)^b^	53 ± 4	53 ± 4	0.94
Stroke volume (mL)	80 ± 20	68 ± 17	0.18
Cardiac output (L/min)	5.7 ± 0.8	5.3 ± 1.3	0.38
Resting regional cerebral perfusion			
All gray matter (mL/min/g)	46 ± 13	45 ± 10	0.88
Cerebellum (mL/min/g)	40 ± 9	36 ± 9	0.33
Frontal lobe (mL/min/g)	51 ± 16	52 ± 12	0.88
Occipital lobe (mL/min/g)	44 ± 14	40 ± 11	0.44
Parietal lobe (mL/min/g)	46 ± 14	45 ± 11	0.92
Temporal lobe (mL/min/g)	42 ± 10	43 ± 9	0.90
Resting cerebral measures in the superior sagittal sinus			
Cerebral blood flow (mL/min)	366 ± 80	381 ± 76	0.67
Cerebral SvO_2_ (%)	79 ± 8	70 ± 8	**0.02**
Cerebral VO_2_ (mL/min)	12.1 ± 5.4	19.5 ± 7.0	**0.02**

*Note*: Data are presented as mean ± SD, with the exception of data that are not normally distributed (Shapiro–Wilk's test), which are presented as median (95% confidence interval).

Abbreviations: BMI, body mass index; BP, blood pressure; BSA, body surface area; EDV, end‐diastolic volume; EF, ejection fraction; ESV, end‐systolic volume; MRI, magnetic resonance imaging; POTS, postural orthostatic tachycardia syndrome; SpO_2_, oxygen saturation; SvO_2_, venous oxygen saturation; VO_2_, oxygen consumption.

Groups were compared using an unpaired Student’s *t*‐test (normally distributed data) or nonparametric Mann–Whitney *U*‐test (non‐normally distributed data). Significance was set a‐priori at *p* < 0.05 and is indicated in bold where applicable.

^a^ Missing values for cardiac MRI due to technical issues (POTS, *n* = 10).

^b^ The RV was unable to be measured in one participant due to imaging difficulties (did not capture entire RV; POTS: *n* = 9).

At rest, there were no differences between patients with POTS and healthy controls in their resting anthropometrics or hemodynamic outcome measures (Table 1). There were also no differences in cerebral perfusion (gray matter of the whole brain, cerebellum, or the frontal, occipital, parietal, or temporal lobes), nor in resting CBF in the superior sagittal sinus; however, cerebral SvO_2_ was lower in patients with POTS, which resulted in higher resting cerebral VO_2_ (Table 1). During the supine resting two‐back test response times were similar between patients with POTS and controls (586 ± 70 and 601 ± 57 ms, respectively; *p* = 0.39); however, the POTS group had a trend to fewer correct responses (75 ± 15 vs. 88 ± 13%; *p* = 0.07).

**FIGURE 3:**
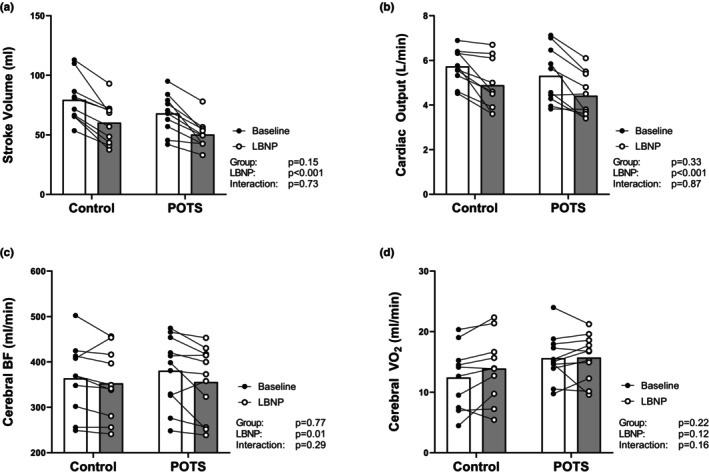
Changes in cardiac and cerebrovascular function during 20 min of −25 mmHg lower body negative pressure (LBNP) in healthy controls (open symbols) and patients with postural orthostatic tachycardia syndrome (POTS; filled symbols). (a) stroke volume, (b) cardiac output, (c) cerebral blood flow (BF), and (d) cerebral oxygen utilization (VO_2_).

Compared to rest, systolic, diastolic, and mean arterial BP were not different during the last 5 min of LBNP (main effect of LBNP: *p* = 0.22, 0.16, and 0.13, respectively). Moreover, BP responses to LBNP were not different between groups (interaction effect: *p* = 0.41, 0.80, and 0.52, respectively). Indeed, mean arterial BP changed +3 ± 6 mmHg and +1 ± 3 mmHg in the POTS and control groups, respectively. Biventricular EDV and ESV decreased during LBNP (*p* < 0.001 for all). This resulted in a decrease in SV and Qc that was comparable between both groups (Figure 3). The magnitude of the decrease was larger for EDV than ESV (Table S1) and thus left and right ventricular ejection fraction also decreased during LBNP (main effect of LBNP: *p* < 0.001 for both).

Regional measures of cerebral perfusion assessed by ASL were decreased during LBNP in the gray matter of the whole brain (main effect of LBNP: *p* < 0.001) but not in the cerebellum (main effect of LBNP: *p* = 0.12). Cerebral perfusion also decreased during LBNP in the frontal, occipital, parietal, and temporal lobes (*p* < 0.01 for all; Table S2). There were no interactions suggesting differential responses between groups for the ASL measures of cerebral perfusion (*p* > 0.05 for all). Similarly, CBF and SvO_2_ in the superior sagittal sinus also decreased during LBNP (*p* = 0.01 and *p* = 0.02, respectively). SpO_2_ also decreased during LBNP (−1% in both groups; main effect of LBNP; *p* < 0.01). Cumulatively, this resulted in no net change in cerebral VO_2_ and there were no differences between the two groups in the cerebrovascular response to LBNP (Figure 3).

During LBNP, more patients with POTS experienced at least one symptom (*n* = 9; 82%) compared to controls (*n* = 2; 20%) and had a higher VOSS (25 ± 21 vs. 5 ± 12; *p* = 0.02). One patient in the POTS group experienced brief loss of consciousness during LBNP. HR increased in both groups during LBNP (main effect of LBNP: *p* < 0.001) with no difference between groups (control: 9 + 8 bpm and POTS: 13 ± 8 bpm; interaction effect: *p* = 0.33). The mean response time and percent of correct responses for the two‐back test were not different during LBNP compared to rest in either group (*p* = 0.80 and 0.94, respectively). Lastly, there were no correlations between VOSS during LBNP and any of our outcome measures within the patient group (Table S2).

## 4 DISCUSSION

The major novel findings for this study were that, contrary to our hypothesis, we did not observe a difference between patients with POTS and healthy controls in the cardiac or cerebral response to 20 min of −25 mmHg LBNP. We also observed decreases in biventricular volume, Qc, cerebral perfusion, and CBF during LBNP in both groups, with a greater increase in VOSS in the patients with POTS; mean arterial BP and cerebral VO_2_ were unchanged during this stimulus. These findings highlight that in individuals with POTS, the cardiac and cerebrovascular response to a moderate level of LBNP is not impaired.

### 4.1 Impact of POTS on cardiac function at rest and in response to LBNP

Previously, Fu et al. (2010), using CMR, reported that resting left ventricular mass was smaller, but EDV was not different in patients with POTS compared to controls. To date, no study has used CMR to comprehensively quantify cardiac volumes, ejection fraction, SV, and Qc in patients with POTS during a simulated orthostatic challenge. Contrary to our hypothesis, we did not observe a difference in cardiac function or volumes at rest between our two groups, although there was a trend for lower right ventricular EDV in the POTS group. It may be that as we primarily recruited young (15‐ to 19‐year‐old) female participants and that the effects of “detraining” due to their symptoms have not yet resulted in marked cardiac atrophy. It remains to be determined if these null observations would hold true in males with POTS, or in older individuals with POTS and future studies should consider age and sex in their study design and analysis.

We previously used CMR to measure the cardiac responses to −30 mmHg LBNP in normally active males and reported a decrease in left ventricular EDV, ESV, SV, ejection fraction, and Qc; the responses we observed for both groups in the present study are similar in magnitude. In the current study we did not report evidence of differences between groups in cardiac function in response to LBNP. This suggests that the level of LBNP stimulus was insufficient to simulate the hemodynamic stress of the standing position and differentiate between groups. From a safety and feasibility perspective, we chose a conservative −25 mmHg stimulus given our patients' history coupled with the need to undergo LBNP inside the MRI. While this stimulus may not reflect the full blood volume shifts induced by standing (Taneja et al., 2007), we did elicit significant changes in biventricular volume for all participants. Moreover, patients did experience symptoms consistent with orthostasis (e.g., headache, light headedness, and nausea) Despite an increase in HR in all participants during LBNP, there was no difference in the HR or BP response for the patients with POTS compared to controls, and only one participant experienced an increase in HR ≥30 bpm. Future studies measuring cardiac (and cerebral) responses to LBNP in patients with POTS may need to utilize greater negative pressure to detect a differential response.

The lower Qc observed at rest in patients with POTS persists during a tilt‐test orthostatic challenge (Fu et al., 2010). In that study, the change in Qc did not appear to be different between groups (control: −2.6 L/min [38%] vs. POTS: −1.8 L/min [36%]) and a group × tilt interaction was not reported. In our study, in response to LBNP the change in Qc was smaller (~16% decrease) which is likely reflected by the modest magnitude of LBNP used. Exercise training for patients with POTS has been reported to increase blood volume and cardiac mass (Fu et al., 2010; Fu & Levine, 2018) and reduced the percentage of adult patients who met the criteria for POTS. We did not account for fitness or habitual physical activity in the present study; however, these should be considered in future studies in POTS.

### 4.2 Impact of POTS on cerebrovascular function at rest and in response to LBNP

Previous studies measuring resting cerebral blood velocity using transcranial Doppler ultrasound report no difference in patients with POTS compared to healthy adults (Lin et al., 2020; Ocon et al., 2012; Stewart et al., 2015; Stewart & Medow, 1985; Wells et al., 2020). However, the limitations of transcranial Doppler include the inability to measure volumetric flow or provide information about cerebral oxygen consumption (Willie et al., 2011). We observed higher resting cerebral VO_2_ in the POTS group, which was unexpected. We believe this baseline finding may have been due to an overestimation of Hgb as some (Antiel et al., 2011; Jarjour & Jarjour, 2013), but not all (Fu et al., 2010), previous research suggests that young females with POTS may suffer from low Hgb. If true in our cohort, this would have resulted in lower cerebral VO_2_ and no differences between. Regardless, our research question was to determine if the responses to LBNP were different between groups, thus our null findings are still valid.

The current hypothesis is that the cerebral symptoms experienced by patients with POTS are associated with altered cerebrovascular responsiveness. Indeed, studies using oximetry to measure cerebral tissue saturation in response to orthostatic challenge have suggested an altered response in patients with POTS (Kharraziha et al., 2019; Tanaka et al., 2002), but no study to date has demonstrated differences in the response to tilt between patients with POTS and controls using transcranial Doppler ultrasound (Esch et al., 2010; Lin et al., 2020). In this regard, our findings align with, and extend, the previous research. Interestingly, Stewart et al (Stewart & Medow, 1985) observed a larger decrease in cerebral blood velocity in patients with POTS who have an exaggerated increase in ventilation upon standing implying that there is a subset of individuals with POTS who may exhibit impaired cerebrovascular response to orthostatic stress. Unfortunately, due to the technical limitations of the MRI environment we did not measure ventilation or carbon dioxide levels during the protocol. However, the lack of difference we observed for cerebrovascular outcomes would support minimal differences in the respiratory response between groups. While we cannot discount the possibility of a Type II error due to the small sample size, these data represent the first measures of their kind in this population and extend the previous findings in the suggestion that there are only minimal differences in cerebrovascular health between individuals with POTS and healthy controls.

### 4.3 Impact of POTS on symptoms of orthostatic intolerance and cognitive function

We used LBNP in an effort to mimic orthostatic challenge and elicit symptoms of cerebral hypoperfusion including light‐headedness, nausea, and syncope. During a cognitive stress test, patients with POTS have been previously reported to have a reduction in cerebral blood velocity that corresponded with symptom score (Esch et al., 2010) and a decline in cognitive function in patients with POTS that is most pronounced upon standing (Beaudry et al., 2018; Thompson et al., 2016). We observed that the POTS group had a greater increase in their symptom score during LBNP compared to controls, but that it was not related to physiological outcomes suggesting that the symptomology and physiology are not as related as we may have expected. Moreover, despite having a lower score on the two‐back test (% correct) compared to the control group at rest, LBNP did not change two‐back test performance nor result in differential decreases in regional cerebral perfusion between the two groups. More comprehensive cognitive function testing with concurrent physiological measures is needed to fully elucidate the mechanistic pathways involved in the symptomology observed in this vulnerable patient population.

### 4.4 Conclusion

Our data suggest that young, primarily female, individuals with POTS who are otherwise healthy appear to have intact cardiac, cerebral, and cognitive function during LBNP. This highlights that the symptoms observed in individuals with POTS may not be driven by significant cardiac or cerebrovascular dysfunction. Future research is necessary to continue exploring mechanisms responsible for the symptoms experienced by this patient group during orthostatic challenge in order to best facilitate the development of disease management strategies.

## FUNDING INFORMATION

This project received funding through the Woman and Children's Health Research Institute (RES0040712).

## CONFLICT OF INTEREST STATEMENT

None.

## ETHICS STATEMENT

This project received ethics approval through the University of Alberta research ethics board (protocol Pro00081704) and conforms to the ethical principles outlined in the Declaration of Helsinki.

## Supporting information


**Data S1:** Supporting information.

## Data Availability

Original data are available upon reasonable request. Detailed methods and additional results can be found in the O*nline Supplement* at doi: https://figshare.com/articles/online_resource/POTS_LBNP‐_Online_supplement/25000346.
