# Supplementary material for: Effect of lower body negative pressure on cardiac and cerebral function in postural orthostatic tachycardia syndrome: A pilot MRI assessment
Source: Physiol Rep. 2024 Mar 15;12(6):e15979. doi: 10.14814/phy2.15979 (PMC10942852; doi:10.14814/phy2.15979)
Supplement: Supplementary file 1 — Data S1: Supporting information. [file PHY2-12-e15979-s001.docx]

**Detailed magnetic resonance (MR) imaging methods:**

*Cardiac Function:*

Cardiac volumes were measured during free-breathing image acquisition as previously described [1-3]. Briefly, using an 18-channel body array coil, image acquisition parameters were as follows: 1.5ms echo time, 60° flip angle, 10-12 slices that were 8mm thick, matrix = 256 x 160, 300-380mm field of view, and a 30-phase temporal resolution. Left and right ventricular end diastolic and systolic volume (EDV and ESV, respectively) were determined from manual endocardial contours traced on the short axis slices at end-expiration using custom Matlab software (RightVol, Belgium). Contours were drawn in reference to 2-chamber and 4-chamber images to aid in accurate identification of the atrio-ventricular plane. LV and RV ejection fraction was calculated as stroke volume ÷ EDV. For the overall hemodynamic responses, stroke volume was defined as the average of left and right ventricular stroke volume, and this was multiplied by heart rate to calculate cardiac output.

*Arterial spin labelling (ASL; regional cerebral perfusion):*

Cerebral perfusion images were acquired using 64 channel head/neck coil. The protocol included a 3-dimensional T1 weighted anatomical scan (sagittal magnetization prepared rapid acquisition gradient echo) that consisted of 208 slices acquired with the following parameters: 1800ms repetition time, 2.37ms echo time, 900ms inversion time, 250x250mm field of view in 3:39min, 0.87x0.87x0.85mm^3^. This was followed by an ASL scan (multiband echo plannar imaging with pseudo-continuous ASL) as implemented by Center for Magnetic Resonance Research, University of Minnesota [4]. The parameters for the ASL were as follows: axial oblique orientation with 3580ms repetition time, 19ms time to echo, multi-band factor = 6, Labeling Duration = 1500 ms, Postlabeling delay = 1641.86 ms, 2.5x2.5x2.3 mm^3^, 60 slices with 0.2 mm gap, 215x215 mm field of view in 5:29 min, 43 pairs of volumes with/without tag, 2 volumes of M0, TI (volumes) = 1700 (6), 2200 (6), 2700 (6), 3200 (10), 3700 (15) ms. Additionally, spin-echo field map was acquired with two opposite phase encoding directions. T1 images were processed using FMRIB software library (FSL; version 5.0.11 [5-7]. Processing script fsl_anat was edited to use 1mm Montreal Neurological Institute (MNI) template instead of standard 2mm template. Outputs used for further analysis were grey matter segmentation and inverse transformation matrix from MNI space to image space. Binary brain grey matter mask was created from probabilistic grey matter images by thresholding only voxels > 90%. For definition of brain lobes as region of interests, FSL’s MNI structural atlas [8, 9] (25% threshold) was transformed from MNI space to individual T1 space. Cerebral perfusion maps were calculated from ASL images using BASIL toolset (oxford_asl version 4.0.5) [10]. White-paper mode was used for all data. Fieldmaps were pre-processed by top up and geometrical distortion correction was applied to all acquired ASL images. Two volumes of M0 were first motion corrected using FSL’s mcflirt, and then averaged. This averaged volume was then used as a reference for motion correction of all ASL volumes. Custom brain mask was created using bet from averaged M0 volume. Perfusion weighted volume was then created by averaging control and tagged volumes first and subtracting the averages using asl_file function. Finally, perfusion maps were created using oxford_asl function. Since T1 volume was used as anatomical reference in oxford_asl processing, one of the outputs was perfusion map aligned and resliced to T1 native space. This map was used for further analysis. First, cerebral perfusion map was masked with binary grey matter mask to retrieve cerebral blood flow (CBF) map in grey matter only. For extraction of cerebral perfusion values in individual brain lobes, MNI atlas in transformed to T1 space was used.

*Cerebral blood flow and venous saturation:*

A custom MR pulse sequence was used for simultaneous measurement of CBF and venous oxygen saturation (SvO_2_) in a time-series of 50 images acquired over 84 seconds (1.68 seconds per time point) with an in-plane spatial resolution of 1.18mm. Briefly, a phase-contrast approach was used to calculate blood velocity maps and a field-mapping method was used to estimate the concentration of deoxyhemaglobin based on the shift in the magnetic field, Δ*B*, within the vein (25533515). Venous oxygen saturation is calculated from Δ*B* according to $Hb0_{2}\left( \% \right)=100\cdot(1-2\cdot\Delta B/(X_{do}\cdot Hct\cdot B_{0}\cdot({cos}^{2}\theta-1/3)))$, where *X_do_* = 4π*0.27 ppm is the susceptibility difference between fully deoxygenated and fully oxygenated red blood cells and *B_0_* is the main magnetic field strength (20408238). Imaging slices were prescribed perpendicular to the long axis of the vein to ensure blood flow is primarily in the through-plane direction. The magnetic field shift in the vein was referenced to a region of interest in the brain that was drawn around the superior sagittal sinus, excluding the skull or other visible blood vessels (**Figure 3**). A custom region growing approach was used to select the pixels within the vein with minimal user input for calculation of CBF (average velocity in all pixels multiplied by the pixel cross-sectional area) and SvO_2_. All analysis was performed in Matlab (v2023a).

**Additional Results:**

**Supplement Table 1. Change in cardiac and cerebral function during LBNP**

|  | Control (n=10) | POTS (n=11) | Interaction  *P*-value^#^ |
| --- | --- | --- | --- |
| **Cardiac measures*** | | | |
| ∆Left ventricular EDV (ml) | -28 ± 11 | -25 ± 14 | 0.57 |
| ∆Left ventricular ESV (ml) | -8 ± 5 | -7 ± 5 | 0.47 |
| ∆Right ventricular EDV (ml)** | -27 ± 14 | -24 ± 16 | 0.86 |
| ∆Right ventricular ESV (ml)** | -8 ± 5 | -7 ± 6 | 0.74 |
| ∆Stroke volume (ml) | -19 ± 10 | -18 ± 10 | 0.79 |
| ∆Cardiac output (L/min) | -0.8 ± 0.7 | -0.9 ± 0.7 | 0.73 |
| **Regional cerebral perfusion** | | | |
| ∆All grey matter (ml/min/g) | -4.9 ± 3.0 | -4.0 ± 4.6 | 0.63 |
| ∆Cerebellum (ml/min/g) | -4.0 ± 5.4 | -0.4 ± 6.6 | 0.21 |
| ∆Frontal lobe (ml/min/g) | -5.0 ± 3.36 | -5.4 ± 5.7 | 0.85 |
| ∆Occipital lobe (ml/min/g) | -4.6 ± 3.5 | -5.5 ± 3.6 | 0.12 |
| ∆Parietal lobe (ml/min/g) | -4.3 ± 2.8 | -5.1 ± 5.2 | 0.67 |
| ∆Temporal lobe (ml/min/g) | -5.5 ± 3.2 | -3.9 ± 4.5 | 0.35 |
| **Cerebral measures in the superior sagittal sinus** | | | |
| ∆Cerebral blood flow (ml/min) | -17 ± 13 | -25 ± 32 | 0.29 |
| ∆Cerebral SvO_2_ (%) | -2 ± 3 | -2 ± 5 | 0.41 |
| ∆Cerebral VO_2_ (ml/min) | 1.1 ± 2.0 | -0.2 ± 3.7 | 0.16 |

Data are presented as mean ± SD. LBNP, lower body negative pressure; POTS, postural orthostatic tachycardia syndrome; EDV, end-diastolic volume; ESV, end-systolic volume; SvO_2_, venous oxygen saturation; VO_2_, oxygen consumption. Regional cerebral perfusion was determined using arterial spin labelling whereas cerebral blood flow in the superior sagittal sinus were determined by phase contrast magnetic resonance (MR) imaging. ^#^*P*-values represent the interaction effect from the repeated measures ANOVA performed on absolute data at supine baseline and during LBNP. *Missing values for cardiac MR due to technical issues (POTS, n=10). **The right ventricle was unable to be measured in one participant due to imaging difficulties (did not capture entire ventricle; POTS: n=9). Significance was set *a-priori* at p<0.05.

**Supplement Table 2. Correlations between symptoms during LBNP (VOSS score) and physiological outcome measures for patients with POTS.**

|  | Number of participants | Pearson correlation coefficient (r) | P-value |
| --- | --- | --- | --- |
| **Cardiac measures during LBNP*** | | | |
| Heart rate (bpm) | 11 | -0.053 | 0.877 |
| MAP (mmHg) | 11 | 0.494 | 0.122 |
| Left ventricular EDV (ml) | 10 | -0.408 | 0.242 |
| Left ventricular ESV (ml) | 10 | -0.351 | 0.321 |
| Right ventricular EDV (ml)** | 9 | -0.297 | 0.439 |
| Right ventricular ESV (ml)** | 9 | -0.364 | 0.336 |
| Stroke volume (ml) | 10 | -0.389 | 0.267 |
| Cardiac output (L/min) | 10 | -0.396 | 0.258 |
| **Cerebral measures during LBNP** | | | |
| ASL - all grey matter (ml/min/g) | 10 | -0.349 | 0.324 |
| ASL - cerebellum (ml/min/g) | 10 | -0.067 | 0.854 |
| ASL - frontal lobe (ml/min/g) | 10 | -0.365 | 0.301 |
| ASL - occipital lobe (ml/min/g) | 10 | -0.123 | 0.735 |
| ASL - parietal lobe (ml/min/g) | 10 | -0.470 | 0.170 |
| ASL - temporal lobe (ml/min/g) | 10 | -0.426 | 0.220 |
| SSS CBF (ml/min) | 11 | -0.244 | 0.504 |
| SSS SvO_2_ (mmHg) | 11 | -0.137 | 0.189 |
| SSS VO_2_ (ml/min) | 11 | -0.076 | 0.201 |

LBNP, lower body negative pressure; VOSS, Vanderbilt orthostatic symptom score; POTS, postural orthostatic tachycardia syndrome; MAP, mean arterial blood pressure; EDV, end-diastolic volume; ESV, end-systolic volume; ASL, arterial spin labelling; SSS, superior sagittal sinus; CBF, cerebral blood flow; SvO_2_, venous oxygen saturation; VO_2_, oxygen consumption. *Missing values for cardiac MR due to technical issues (POTS, n=10). **Additionally, the right ventricle was unable to be measured in one participant due to imaging difficulties (did not capture entire ventricle; POTS: n=9). Relationships between VOSS and cardiac or cerebral outcomes during LBNP were determined by Pearson’s correlation coefficient (r) and significance was set *a-priori* at p<0.05.

**Supplement References:**

1. Beaudry, R.I., et al., *Exercise cardiac magnetic resonance imaging: a feasibility study and meta-analysis.* Am J Physiol Regul Integr Comp Physiol, 2018. **315**(4): p. R638-r645.

2. Thompson, R.B., C.R. Tomczak, and M.J. Haykowsky, *Evaluation of Cardiac, Vascular, and Skeletal Muscle Function With MRI: Novel Physiological End Points in Cardiac Rehabilitation Research.* Can J Cardiol, 2016. **32**(10 Suppl 2): p. S388-s396.

3. Esch, B.T., et al., *Changes in ventricular twist and untwisting with orthostatic stress: endurance athletes versus normally active individuals.* J Appl Physiol (1985), 2010. **108**(5): p. 1259-66.

4. Li, X., et al., *Theoretical and experimental evaluation of multi-band EPI for high-resolution whole brain pCASL Imaging.* Neuroimage, 2015. **106**: p. 170-81.

5. Jenkinson, M., et al., *FSL.* Neuroimage, 2012. **62**(2): p. 782-90.

6. Smith, S.M., et al., *Advances in functional and structural MR image analysis and implementation as FSL.* Neuroimage, 2004. **23 Suppl 1**: p. S208-19.

7. Woolrich, M.W., et al., *Bayesian analysis of neuroimaging data in FSL.* Neuroimage, 2009. **45**(1 Suppl): p. S173-86.

8. Collins, D.L., et al., *Automatic 3-D model-based neuroanatomical segmentation.* Human Brain Mapping, 1995. **3**(3): p. 190-208.

9. Mazziotta, J., et al., *A probabilistic atlas and reference system for the human brain: International Consortium for Brain Mapping (ICBM).* Philos Trans R Soc Lond B Biol Sci, 2001. **356**(1412): p. 1293-322.

10. Chappell, M.A., et al., *Variational Bayesian Inference for a Nonlinear Forward Model.* IEEE Transactions on Signal Processing, 2009. **57**(1): p. 223-236.
